# Case Report: Diagnostic assessment, developmental trajectory and treatment approaches in a case of a complex neurodevelopmental syndrome associated with non- synonymous variants in *MECP2* (p. R133C) and *GABBR1*

**DOI:** 10.3389/fped.2025.1617479

**Published:** 2025-06-19

**Authors:** V. Napoli, S. Guerrera, F. Demaria, G. Piccolo, A. Cianfa, S. Passarini, M. G. Logrieco, G. Zanni, G. Valeri, S. Vicari

**Affiliations:** ^1^ Child and Adolescent Neuropsychiatry Unit, Bambino Gesù Children’s Hospital, IRCCS, Rome, Italy; ^2^ Child Neurology and Psychiatry Unit, Fondazione Policlinico Universitario Agostino Gemelli IRCCS, Rome, Italy; ^3^ Laboratory of Medical Genetics, Translational Cytogenomics Research Unit, Bambino Gesù Children’s Hospital, IRCCS, Rome, Italy; ^4^ Department of Biomedicine and Prevention, Tor Vergata University of Rome, Rome, Italy; ^5^ Department of Dynamic and Clinical Psychology and Health Studies, Sapienza University of Rome, Rome, Italy; ^6^ Department of Humanites, University of Foggia, Foggia, Italy; ^7^ Rare Diseases and Medical Genetics Unit, Bambino Gesù Children’s Hospital, IRCCS, Rome, Italy; ^8^ Life Sciences and Public Health Department, Catholic University, Rome, Italy

**Keywords:** Rett syndrome, methyl cpG binding protein 2, Zappella Rett syndrome clinical variant (Z-RTT), preserved speech variant (PSV), GABAB, intellectual disability, tics and Tourette syndrome

## Abstract

**Background:**

Rett Syndrome (RTT) is an X-linked progressive disease affecting 1 in 10,000 females. *MECP2* p.R133C, is the second most common variant affecting more than 4% of all RTT cases. *GABBR1* pathogenic variants have been recently associated with mild to severe psychomotor delay, epilepsy, intellectual disability (ID), autism (ASD), attention deficit hyperactivity disorder (ADHD) and oppositional defiant disorder (ODD).

**Material and methods:**

We report a 13.9-year-old girl, with a complex neurodevelopmental disorder including ASD, ID with the appearance, at 9 years of age, of vocal and motor tics involving the upper limbs and trunk, suggesting a diagnosis of Tourette's syndrome (TS). Tics were also present in the mother and grandmother. The patient was followed-up for approximately 10 years and underwent periodic clinical and neuropsychological evaluations. We performed Trio-based WES analysis and segregation analysis in relevant family members.

**Results:**

A *de novo MECP2* variant (p. R133C) was detected in the proband. Moreover, a maternally inherited VoUS class 3 in *GABBR1* (p. F692S), was identified in the proband, and segregated in the mother and grandmother. No functional studies confirm the pathogenicity of this *GABBR1* variant, and Tourette phenotypes have not been previously linked to *GABBR1*. Based on familial segregation, we hypothesize that this variant may worsen the *MECP2*-related phenotype and underlie the Tourette symptoms seen in all carriers. Tourette phenotypes have never been reported with *MECP2* variants alone. Although Rett-like features are mainly due to *MECP2* loss-of-function, *MECP2* deficiency disrupts GABAergic signaling, making GABA modulators potential therapeutic targets. The presence of the *GABBR1* variant may further impair GABA receptor neurotransmission. Thus, the *GABBR1* variant may be a modifying factor in this case, though its pathogenicity remains uncertain. Despite attempts to manage her condition with appropriate pharmacological therapies, progressive muscle hypertonia and behavioral issues, persisted. The patient showed improvements in engagement and emotional regulation, during music therapy sessions.

**Conclusion:**

We describe the developmental trajectory of an adolescent with overlapping features of Rett and Tourette syndromes, carrying *MECP2* and *GABBR1* variants. Future studies are essential to better characterize the genotype-phenotype correlates and optimize therapeutic strategies, to tackle the unique needs of the patient and her family.

## 1 Introduction

Rett syndrome (RTT) is a severe neurodevelopmental disorder (1) affecting approximately 1 in every 10,000–15,000 live female births, caused by variant in the *MECP2* gene on chromosome Xq28, which encodes a protein essential for transcriptional regulation and the development and plasticity of the central nervous system (2). Genotype–phenotype correlation studies have shown that while large deletions in *MECP2* are linked to the most severe forms of RTT, specific missense variant (e.g., R133C, T158M, and R306C) account for roughly 25% of cases and are generally associated with milder clinical presentations (3).Clinically, RTT is classified into typical (classic) and atypical (variant) forms (4). Among the atypical subtypes, the Zappella variant—also known as the Preserved Speech Variant (PSV) (Z-RTT)—is most frequently associated with the R133C variant. This variant is marked by a delayed regression phase and milder deficits in hand function and intellectual abilities. R133C variant is associated with a milder clinical presentation, characterized by improved ambulation, better hand function, and a higher likelihood of speech acquisition (5) (See Supplementary Table S1). To our knowledge, the most recent studies detailing the clinical profiles of Z-RTT/PSV date back to 2016 (6).

We present the case of a 13.9-year-old female patient with the p.R133C variant of RTT, who has been followed for over 10 years at the Child and Adolescent Psychiatry Unit of Bambino Gesù Children's Hospital, IRCCS. This case report aims to describe her developmental trajectory, clinical features, and the therapeutic approaches implemented over the years.

## 2 Case report

F. is the firstborn of non-consanguineous parents, he pregnancy was uneventful except for a threatened miscarriage during the first trimester, managed with maternal rest. She was born at 41 weeks by dystocic delivery with vacuum extraction; growth parameters at birth were normal (3,170 g; head circumference 35 cm). Her parents reported that early motor milestones were achieved on time (head control by 3 months, trunk control by 6 months, and independent walking at 14 months). Clinical symptoms began at 24 months, when F. exhibited social withdrawal, reduced eye contact, difficulties in relationships, and limited verbal communication. Hand stereotypies (e.g., midline pinching and hand clapping) were evident from the initial evaluation. Consequently, she underwent an outpatient assessment at our center, and at 3 years of age (in 2014) she received a diagnosis of autism spectrum disorder. At age 5, exome analysis identified a variant in the *MECP2* gene, confirming a diagnosis of Z-RTT/PSV. Around the age of 9, the patient began showing vocal and complex motor tics, mainly affecting the upper limbs and trunk. These movements appeared distinct from her pre-existing autistic stereotypies and led to a clinical diagnosis of TS. TS is a neurodevelopmental disorder characterized by sudden, repetitive, non-rhythmic motor and vocal tics (1), typically emerging in school-age children and affecting approximately 0.5% of those aged 4–18 (7). Diagnosis requires the presence of multiple motor tics and at least one vocal tic, persisting for more than one year (8). About a year after the onset of tics, she developed anxiety symptoms. By the age of 13.5, self-injurious behaviors such as skin picking had appeared. Currently, she presents with toe walking, broad-based gait, and progressive hypertonia with rigidity of the limbs. She has moderate intellectual disability, limited verbal output, and worsening hand stereotypies that have impaired her fine motor abilities and made the use of augmentative and alternative communication (AAC) has become increasingly challenging.

**Figure 1:**
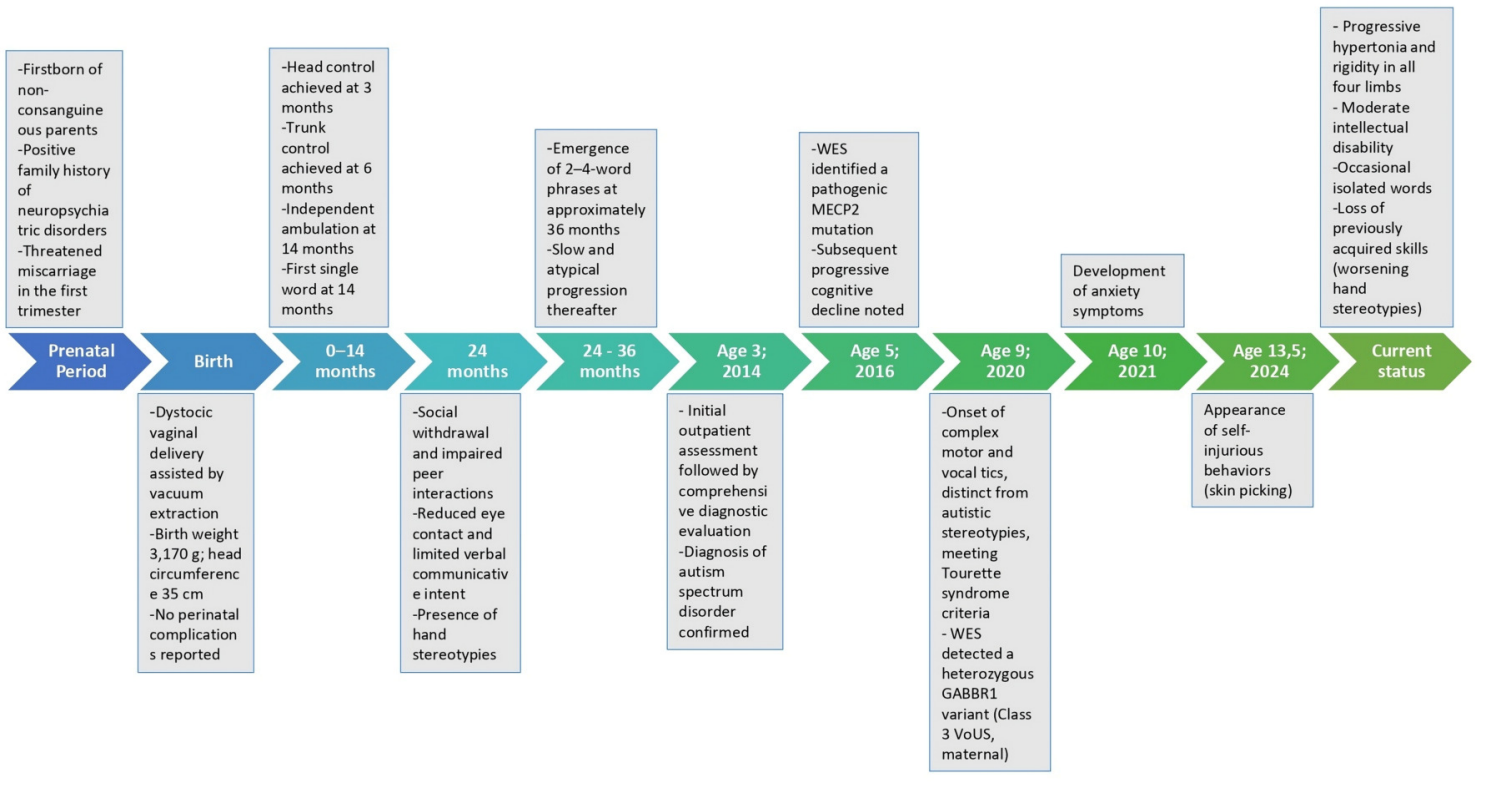
Chronological clinical history of the patient. The timeline illustrates the main developmental milestones, clinical features, diagnostic evaluations, and genetic findings from the prenatal period to age 13.5 years in the patient described in the case report.

Figure 1 illustrates the timeline of F.'s clinical history.

### 2.1 Family history (carrier phenotype)

F. comes from a family with a significant history of neuropsychiatric disorders: the maternal lineage presents a positive family history of TS, anxiety disorders, and obsessive–compulsive disorder, whereas the paternal side exhibits mood disorders. The patient's mother has a confirmed diagnosis of Tourette's syndrome and is under ongoing specialist follow-up for this condition. Although she has never undergone standardized neuropsychological assessments, the available information was collected through an anamnestic reconstruction of her developmental history. A formal cognitive profile is not available, but she attained a university degree without reporting any academic or adaptive difficulties. Her neuropsychiatric profile during childhood was characterized by sensory peculiarities related to feeding (e.g., smelling food), although no marked selectivity or other related features were reported. During the same period, she exhibited compulsive behaviors and an anxiety condition associated with emotional distress and psychological stress. The exact age of symptom onset was difficult to determine. Also in childhood, she developed a compulsive need to pull or break her hair, consistent with trichotillomania. It was not possible to specify whether these behaviors were accompanied by trichophagia, although it was not excluded. She denied the presence of obsessive thoughts or stereotyped motor behaviors. During adolescence, she reported significant anxiety and panic attacks, along with sleep disturbances. At that time, motor tics such as eye blinking and trunk twisting movements were present, later accompanied by vocal tics described as guttural sounds and coughing. The severity of symptoms warranted psychopharmacological treatment with aripiprazole and mirtazapine. In adulthood, symptoms attenuated, although a marked anxious trait persisted.

Moreover, we collected anamnestic information regarding the patient's maternal grandmother, which revealed a similar developmental profile. This included sensory peculiarities related to feeding during childhood, the presence of tics throughout development, a generalized anxiety condition, and a more pronounced externalizing profile characterized by impulsivity and motor restlessness. It is important to note that the grandmother has never undergone formal standardized evaluations. It is not possible to know the presence of cognitive, adaptive or relational impairments (See Supplementary Table S2).

### 2.2 Genetic analysis

#### 2.2.1 Exome sequencing

Genomic DNA was extracted from peripheral blood of the girl and her parents, using commercial kit. Informed consent was obtained from all participating subjects according to the Declaration of Helsinki. Whole exome sequencing was performed using Illumina HiSeq X, and the resulting 150 bp paired-end reads were aligned to the GRCh38 reference genome. Data analysis was performed using an in-house implemented pipeline, mainly based on the Genome Analysis Toolkit (GATK v3.7). To prioritize variants, we applied a sequential filter to retain only those variants with the following characteristics: (a) potential effect on protein and transcript; (b) consistency with the suspected inheritance model (*de novo* or autosomal recessive); and (c) consistency with a neurodevelopmental/neurological phenotype.

Exome sequencing detected a known heterozygous *de novo* missense variants in *MECP2* NP_004983.1: c.397C>T; p.Arg133Cys (rs28934904) in the patient. Additionally, exome identified a maternally-inherited heterozygous variant (NM_001470.4) c.2075T>C; p.Phe692Ser (rs780587551) in *GABBR1* classified as VoUS Class 3, according to ACMG Standards and Guidelines, with an allelic frequency of 0.000024 (GnomAD v4.1.0). This variant is located within exon 17 out of 23, causing substitution of a nonpolar aromatic amino acid (phenylalanine), with a polar hydroxylic amino acid (serine). Variant pathogenicity was estimated with SIFT (0.064), PrimateAI (0.89), REVEL (0.85), MutationTaster (0.99), AlphaMissense (0.966), VARITY (0.87), fitCons (0.71), CADD (29.5) prediction scores. Moreover, the F692 residue is located within a conserved site across species (PhyloP100way: 8.792 and Polyphen-2: 0.98), in particular, in the “transmembrane domain 3 (TMD3)”.

#### 2.2.2 Whole genome array-CGH

DNA was also analyzed by CGH-microarray using high resolution Affymetrix SNP- array GeneChip 6.0 to exclude potential pathogenic Copy Number Variations (CNVs). Data were analyzed using the Agilent Cytogenomics software (Agilent Technologies, Santa Clara, CA, USA; Agilent Cytogenomics v3.0.6.6). Whole Genome Array-CGH yielded normal results.

### 2.3 Neuroradiological and neurophysiological assessment

At age 3 (2015), F. underwent an MRI, which revealed no abnormal cerebral signals and normal DWI findings. An EEG performed in both awake and asleep states showed no epileptiform anomalies.

### 2.4 Assessment of medical co-occurrence

Routine laboratory tests over the years showed normal complete blood counts and biochemical parameters. Annual ECGs consistently demonstrated normal sinus rhythm with a QTc < 400 msec. At 9.7 years, F. developed cyclic vomiting, prompting an esophagogastroduodenoscopy (EGDS) with macroscopically normal findings and negative histology. Her current height is between the 50th and 75th percentiles, though her weight is below the 3rd percentile. While head circumference was normal during the first year, the current measurement (51 cm) is below the 3rd percentile (WHO Growth Charts); serial measurements are unavailable.

### 2.5 Neuropsychological and psychopathological assessment

#### 2.5.1 Cognitive and adaptive development

F. was first evaluated at age 3 using the Griffiths Mental Development Scales (9). The GMDS differentiates degrees of motor and cognitive development by measuring how a child's abilities deviate from a reference sample (GMDS-ER 2-8; Luiz et al., 2006), assessing five subscales: Locomotor, Personal–Social, Language, Eye-Hand Coordination, and Performance. At this first evaluation, no significant deficits were detected. From age 4, her cognitive development was monitored over 10 years with six assessments using the Leiter International Performance Scale, with the Leiter-R (10) and Leiter-3 versions (11). The Leiter-3 is a non-verbal intelligence test designed to evaluate cognitive abilities, memory, and attention in individuals without language impairments, providing a non-verbal intelligence quotient (NVIQ). The results showed a progressive decline in NVIQ from age 4–13.8 years (see Table 1). Adaptive functioning, assessed with the Adaptive Behavior Assessment System, Second Edition (ABAS-II) (12) remained below age expectations, with a modest decline of 15 points over these 10 years (See Table 1).

**Table 1:** Assessment tools cognitive and adaptive development.

Date	Age (years)	Test	NVIQ	ABAS/VABS	ABIQ scores
01/02/2014	3.1	GMDS-E	48	VABS	2.3 - 2.3 - 1.1 -<1.6
01/06/2015	4.5	LEITER-R	91	ABAS-II	55-52-49-49
01/05/2016	5.4	LEITER-3	63	ABAS-II	53-60-44-48
11/01/2017	6	LEITER-3	74	ABAS-II	55-60-42-46
01/03/2018	7.2	LEITER-3	66	ABAS-II	42-56-55-40
01/05/2020	9.4	LEITER-3	59	ABAS-II	40-49-54-40
01/09/2024	13.8	LEITER-3	45	ABAS-II	40-<57-50-44

Note: NVIQ, Nonverbal Intelligence Quotient; *, Performance Quotient from the Griffiths Mental Development Scales—Extended Revised (GMDS-E), Performance subscale; GMDS-E, Griffiths Mental Development Scales—Extended Revised; LEITER-R/3, Leiter International Performance Scale—Revised, Third Edition; ABAS, Adaptive Behavior Assessment System; VABS, Vineland Adaptive Behavior Scales; ABIQ, Adaptive Behavior Index Quotients. ABIQ scores are presented in the following order: GAC, general adaptive composite; C, conceptual; S, social; P, practical.

#### 2.5.2 Language development

At age 3, language was evaluated using the Primo Vocabolario del Bambino – MacArthur-Bates Communicative Development Inventories, which indicated a score below the normative range for the patient's chronological age (13).

At 5.4 years, the Test Fono- Lessicale (TFL) (14) revealed a decline in lexical comprehension (<5th percentile) and limited verbal output. From age 7, F. showed gradual language recovery, using short, simple sentences. No formal language tests were given, but she was assessed with the Autism Diagnostic Observation Schedule-Second Edition (ADOS-2) (15), Module 2. By age 11, her parents noticed a decline in language skills. In 2024, at 13.9 years, F. used single words occasionally for requests, but no verbal production was observed during the evaluation. Echolalic speech, previously present, had disappeared.

#### 2.5.3 Autism symptoms

The presence of autism symptoms was further explored using the ADOS-2. The ADOS-2 is a semi-structured direct assessment of communication, social interaction, and play with materials, or imaginative use of materials, for individuals with suspected autism. F. underwent five ADOS-2 assessments over 10 years (initially at 3 years with Module 2, then at 4.5 years with Module 1, followed by three assessments with Module 2). In the most recent evaluation at 13.9 years, Module 1 was attempted but could not be completed; symptoms were then assessed by clinical observation. The calibrated severity scores consistently indicated a moderate-to-severe autism spectrum disorder, especially in reciprocal social interaction, stereotyped language, echolalia, and hand stereotypies, which have worsened over time (See Table 2).

**Table 2:** Assessment autism symptoms.

Date	Age (years)	ADOS-2 module	Autism symptoms
01/02/2014	3.1	2	9/10 CSS
01/06/2015	4.5	1	7/10 CSS
01/05/2016	5.4	2	7/10 CSS
01/03/2018	7.2	2	8/10 CSS
01/09/2024	13.8	1	N/A

Note: ADOS-2, Autism Diagnostic Observation Schedule, Second Edition; CSS, Calibrated Severity Score; N/A, Not Available. Modules were selected based on the patient's language level and developmental profile at the time of assessment. CSS scores range from 1 (minimal evidence of autism-related symptoms) to 10 (high severity). N/A, not applicable; the ADOS-2 Module 1 could not be completed due to the patient's clinical condition.

#### 2.5.4 Assessment of other neurodevelopmental and psychopathological disorders

At the age of 9,4, F. exhibited both simple and complex motor tics (non-rhythmic and afinalistic movements of the upper limbs and trunk twisting movements) and vocal tics (vocalizations and coprolalia) apparently not related to the stereotyped behaviors of autism but susceptible to a clinical diagnosis of TS.

At 13.56 years, frequent emotional and behavioral dysregulation emerged, including self-injurious behaviors (skin picking) and outward aggression, with compulsive skin picking intensifying during emotional stress. At 12 years, sleep–wake irregularities were noted, with difficulties in falling asleep and frequent awakenings.

## 3 Interventions

### 3.1 Psychosocial interventions

Since age 3, F. has undergone behavioral intervention based on the Applied Behavioral Analysis (ABA) model (16) for about 2 years before transitioning to therapy based on the Developmental Individual-Difference Relationship (DIR) model (17). Starting at age 7, she has received ongoing cognitive-behavioral psychoeducational support. Additionally, beginning at age 6, she received speech therapy incorporating augmentative and alternative communication (AAC) tools (18) until she was 9, has consistently participated in water therapy since age 3 (19).

### 3.2 Pharmacological therapy for psychopathological comorbidities

Table 3 provides a detailed overview of pharmacological therapy, since May 2016 to July 2024.

**Table 3:** Pharmacological intervention.

Date	Age (years)	Pharmacological therapy	Dosage	Remarks
May 2016	5.4	Aripiprazole	10 mg/day	Initiated due to repetitive behaviors
May 2017	6.4	Aripiprazole (discontinued)		Reduced due to hyperphagia e loss of clinical efficacy
May 2018	7.4	Buspirone	2.5 mg/day	Initial improvement in motor hyperkinesia
May 2019	7.5	Buspirone (discontinued)		Loss of clinical efficacy
May 2019	8.4	Fluoxetine	10 mg/day	Discontinued due to worsened hyperactivity and irritability
May 2020	9.4	Xenazine	50 mg/day	Marked improvement in hand stereotypies
January 2021	9	Xenazine (discontinued)		Difficulties in obtaining medication
January 2023	12	Quetiapine	50 mg/day	Difficulty falling asleep
July 2024	13.6	Aripiprazole (restarted)	5 mg/day	Restarted due to worsening repetitive movements

## 4 Discussion

This study examines the developmental trajectory, clinical and genetic features, and therapeutic approaches in a young female patient with Z-RTT/PSV, contextualizing these aspects within the existing literature.

The preserved speech variant (Z-RTT/PSV) (6, 20), is generally associated with a milder clinical presentation compared to classic RTT (20). The recurrent p.R133C variant in *MECP2*, found in our patient, has been previously described in individuals with preserved speech and in families with mildly affected members (two mildly affected sisters and an asymptomatic mother) (21).

Zappella et al. analyzed a cohort of females with preserved language, including two with the R133C variant, and proposed that R133C may serve as a predictive marker of a less severe phenotype (20). In this context, our patient exhibits some similarities with the typical presentation of Z-RTT/PSV, specifically in the phenotypic profile most commonly observed in patients with the R133C variant, such as the achievement of motor developmental milestones within expected timelines, early language development leading to verbal communication characterized by combinations of two to three words (6), and the classic hand stereotypies (5, 6). However, our patient also presents certain characteristics that deviate from those described in the “Zappella criteria” and support the phenotypic traits observed in a small percentage of females with of Z-RTT/PSV in the most recent cohort study available in the literature (22). These include the loss of previously acquired language skills, including comprehension (20), more severe intellectual disability with a progressive decline in cognitive abilities over the years (6), autistic traits that appear to be more pronounced and have worsened during adolescence (6), a decrease in head circumference despite normal stature (22), a progressive decline in fine motor skills and the gradual onset of rigidity and hypertonia (22). While these features have been observed in some cases of R133C phenotypes (5) the developmental trajectories of such abilities, particularly their potential regression, have not been described in detail in the literature. These aspects, observed in our patient, are crucial for prognosis and treatment planning. Given these atypical clinical features, not commonly reported in Z-RTT/PSV alone, we further explored the possibility of a modifying genetic mechanism. Although Rett-like phenotypes are primarily driven by MECP2 loss-of-function, it is well established that MECP2 deficiency disrupts GABAergic signaling to such an extent that GABAergic modulators have been proposed as potential therapeutic targets in RTT. Importantly, reduced GABAergic inhibition has been demonstrated in Tourette syndrome (23), supporting the hypothesis that GABAergic dysfunction plays a central role in Tourette pathophysiology. The coexistence of the *GABBR1* p.F692S variant of uncertain significance raises the hypothesis that this allele could further impair GABA_B_ receptor–mediated neurotransmission, thereby exacerbating specific features in our proband—namely late-onset regression, complex motor and vocal tics, skin-picking, and severe behavioral dysregulation—that are not typically seen in Z-RTT/PSV. This model reconciles the dual-genotype concept with our segregation data: the *GABBR1* variant may potentially act additively or as a modifier of the *MECP2* phenotype, intensifying the GABAergic dysfunction already present in the proband. Functional studies are required to validate this interaction.Medical co-occurrence: F. did not present any respiratory or cardiac abnormalities, consistent with what is described in the literature regarding the Z-RTT/PSV (6). However, she has experienced gastroesophageal reflux with cyclic vomiting for approximately two years (in the absence of any organic alterations (EGDS and negative biopsy). This autonomic alteration has been found in less than half of females with Z-RTT/PSV, specifically in those carrying the R133C variant (5), and according to the literature, it presents in a milder form compared to classic forms (22). From a growth perspective, our patient, in accordance with “Zappella criteria”, did not exhibit growth abnormalities during childhood and currently shows height and weight within the appropriate percentiles for her age (6). Head circumference exhibited consistent growth during the first year of life, like all cases of Rett syndrome (22). In the following years, she currently falls below the 3rd percentile, as reported in a small percentage of patients with the R133C variant described in the literature (5). In terms of motor skills, our patient achieved motor development milestones within the typical timeframe, as outlined in the Zappella criteria (6), although she has consistently demonstrated gross motor difficulties (e.g., she is unable to ride a bicycle). Over the past two years, she has exhibited progressive postural rigidity, with the onset of muscle hypertonia. At age 13.9, cogwheel rigidity was noted during the neurological examination. Such features have been observed in females with Z-RTT/PSV carrying the R133C variant, albeit generally to a lesser extent than in classic RTT, with only a very small percentage of patients losing the ability to walk independently (5, 22). In the case of F., the hypertonia and rigidity observed have proven to be highly disabling and noteworthy. Furthermore, consistent with the literature, we did not observe significant scoliosis or kyphosis (6).

Neuropsychological and psychopathological features: Our patient has demonstrated a progressive and homogeneous decline in cognitive abilities (See Table 1). In just under 10 years, F. has lost 46 points in her cognitive level, resulting in a NVIQ of 45, indicating moderate intellectual disability. This characteristic deviates from what females with the clinical variant known as Z-RTT/PSV usually exhibit—a milder cognitive impairment compared to those with typical RTT (22). To the best of our knowledge, no quantitative data on intellectual development have been documented in the literature for individuals with the R133C variant.

Regarding adaptive functioning, to our knowledge, there are no descriptions in the literature in both typical RTT and Z-RTT/PSV. Our patient consistently exhibited levels lower than those corresponding to her cognitive development (See Table 1). Future research is necessary to evaluate other cases of deterioration in acquired skills. From a linguistic perspective, our patient initially exhibited normal language development (4), followed by the characteristic regression observed in Rett syndrome, although this occurred at a later age (after 5 years), consistent with the criteria for the clinical variant of Z-RTT (6). However, our patient experienced a gradual recovery of language abilities starting at age seven, in line with what Zappella described in his criteria for the preserved speech variant (6) and in some clinical cases reported in the literature (24). Based on the available data on the R133C variant, it emerges that most patients retain at least a minimal level of language, although verbal abilities show significant variability (5). However, to our knowledge, the literature does not provide a detailed description of the developmental trajectory and potential regression of these abilities. Unlike most females with the Z-RTT/PSV, our patient began to experience a gradual decline in her language functions from the age of eleven, reaching a point at 13.9 years where she only occasionally used single words, primarily for requests. This significant regression in language has been described in a minority of cases in the literature (25), highlighting that while some patients may show language skill regression, their language comprehension typically remains relatively intact, a characteristic not observed in our patient (25). Concerning the autistic symptomatology F. appears to align with the criteria described by Zappella, which mention autism characterized by an improvement during the latter part of the first decade of life, followed by a significant deterioration (22).

Hands stereotypies a defining feature of RTT (26) worsened since the age of 10, significantly impacting her previously acquired autonomies, such as eating and drinking independently, as well as her usage of AAC strategies. In addition to finger-twisting movements, our patient began exhibiting challenging behaviours, such as self-directed and other-directed skin-picking at the age of 11. Specific cases of skin-picking have not been documented in relation to the clinical variant of Z-RTT/PSV in the literature. Finally, internalizing and externalizing symptoms have significantly impacted her overall functioning since the age of 13. The presence of anxiety and depression has been documented in patients with classic RTT, while externalizing symptoms appear more prevalent in those with greater preserved motor function (27). Literature on the atypical Z-RTT form is limited, although it seems that the intensity of general anxiety is inversely proportional to the clinical severity presented by the patient (22). The emergence of coprolalia and complex motor tics in our patient at age 9.4 (2020) is likely more associated with a family history of TS than with Z-RTT. Genetic testing is underway to confirm this hypothesis.

Psychosocial and pharmacological interventions In line with the literature (28), integrated psychosocial interventions (ABA, DIR) and the use of AAC tools were applied. During the last Day Hospital observation in September 2024, our patient showed increased environmental participation and fewer crying episodes while listening to music. Regarding pharmacological treatment, no recognized effective medications for RTT exist, except for Trofinetide (an analog of the C-terminal domain of insulin-like growth factor 1), which was recently FDA-approved for RTT and is available only in the United States (29).

Several pharmacological attempts were made in our patient, including antipsychotics for emotional and behavioral dysregulation and sleep disturbances (30). Selective serotonin reuptake inhibitors (SSRIs) were also tried, but these resulted in a paradoxical effect (28). Additionally, Xenatina was used for managing coprolalia and tic-like movements, showing benefits in reducing motor and vocal tics as well as repetitive finger-twisting and skin-picking behaviors, although this medication has not been documented for Z-RTT.

## 5 Conclusion

In this clinical case, we analyzed the developmental trajectory of a female patient diagnosed with the Zappella variant of Rett syndrome in 2016, highlighting deviations from the expected course. Advances in genetic research suggest moving beyond broad phenotypic classifications, such as the “Zappella variant,” toward a more precise analysis of individual genetic variants. Even the same variant, such as R133C, can present with different phenotypes, underscoring the need for individualized assessment. Grouping variants into heterogeneous categories may obscure clinically relevant differences. Moreover, our patient carries a recurrent *MECP2* variant and a class 3 VoUS in *GABBR1*, presenting a complex neurodevelopmental disorder with overlapping RTT and TS features. *GABBR1* encodes a subunit of the GABA_B_ _BB​ receptor, essential for neuronal inhibition. Monoallelic *GABBR1* variants have been linked to motor/language delay of varying severity, with one case of epilepsy. Additional features include intellectual disability, learning difficulties, autism, ADHD, oppositional defiant disorder, sleep disturbances, and hypotonia (31–35). In this context, the coexistence of a recurrent *MECP2* variant and a *GABBR1* VoUS in our patient supports the hypothesis of a potential additive or modifying effect of GABBR1, particularly through GABAergic signaling pathways already known to be impaired in MECP2 deficiency. Although functional validation is still lacking, this dual-genotype framework may help explain the atypical features observed and guide more individualized therapeutic strategies. Further studies are needed to clarify genotype-phenotype correlations and refine pharmacological and psychosocial interventions, tailoring them to the patient's and family's needs to improve clinical outcomes.

## Data Availability

The raw data supporting the conclusions of this article will be made available on request from the corresponding authors. The data are not publicly available due to privacy or ethical restrictions.
