# Supplementary material for: Case Report: Diagnostic assessment, developmental trajectory and treatment approaches in a case of a complex neurodevelopmental syndrome associated with non- synonymous variants in MECP2 (p. R133C) and GABBR1
Source: Front Pediatr. 2025 Jun 19;13:1617479. doi: 10.3389/fped.2025.1617479 (PMC12222089; doi:10.3389/fped.2025.1617479)
Supplement: Supplementary file 1 [file Datasheet1.pdf]

Supplementary Table

1 Supplementary Table 1 Comparison of Clinical Features Between Patient S.F. and Reported Cases with R133C Mutation (Leonard et al., 2003)

Characteristic	Patient S.F. (y/n)	R133C (Percentage in Category, Leonard et al., 2003 (n=25)
**Feeding**		
- Uses spoon	Yes	35%
- Finger feeds		20%
- No attempt		45%
**Gross Motor Function**		
- Walks normally	Yes	5%
- Walking impaired		54%
- Previously or has never walked		38%
**Speech**		
- More than single words		9%
- Single words	Yes	48%
- More than no vocalization but no single words		17%
- Lost speech		22%
- Never acquired		4%
**Age at Losing Social Interaction**		
- >18 months	Yes	86%
- 6–18 months		14%
**Sleep Disturbance**		
- No disturbance reported		40%
- Disturbance either past or present	Yes	27%
- Disturbance both past and present		33%
**Hand Use**		
- Acquired and conserved	Yes	29%
- Lost purposefulness 2–6 years or conserved manipulation		29%
- Lost purposefulness <2 years or conserved grasping		33%
- Acquired and lost		10%
- Never acquired		0%
**Respiratory**		
- No dysfunction	Yes	45%

- Hyperventilation and/or apnea		55%
**Disturbed Awake Breathing Rhythm**		
- Never	Yes	53%
- Rare to daily		21%
- Daily to constantly		26%
**Frequency of Hand Stereotypies**		
- Never		5%
- Rare to dominating		21%
- Dominating to constantly	Yes	74%
**Voluntary Hand Use**		
- Normal, feeding independently	Yes	20%
- Some hand use, feeding with help		55%
- None		25%
**Scoliosis**		
- No scoliosis	Yes	65%
- Scoliosis		35%
- Scoliosis operated		0%
**Ambulation**		
- First walked <18 months and still walking	Yes	53%
- First walked <18 months then lost ability, or first walked 18–30 months and still walking		20%
- First walked 18–30 months then lost ability		20%
- First walked >30 months and still walking		7%
- Never acquired		0%
**Epilepsy**		
- Never	Yes	21%
- Previous seizures, or present and controlled with medication		74%
- Uncontrolled		0%
- Early epilepsy <12 months		5%
**Present Weight (Z score)**		
- Z score >= -1	Yes	59%
- -2 < Z score < -1		6%
- Z score <= -2		35%
**Early Developmental Progress**		
- Normal progress	Yes	61%
- Suboptimal progress		39%
- No or virtually no progress		0%

**Present Head Circumference (HC)**		
- Above 10th centile		53%
- 3rd–10th centile		26%
- Below 3rd centile	Yes	21%
**Present Height (Z score)**		
- Z score >= -1	Yes	25%
- -2 < Z score < -1		31%
- Z score <= -2		44%
**Age at Sitting Alone**		
- Acquired <8 months	Yes	77%
- Acquired 8–16 months		23%
- Never acquired		0%

Supplementary Table 2 Phenotypic Expression of Neuropsychiatric Features in the Proband and Her Family

**Symptoms**	**F. (our patient)**	**Mother**	**Grandmother**
Compulsive behavior	++	++	-
Motor/vocal tics	++	++	+
Anxiety	+	++	++
Sensoriality	++	+	+
Stereotypies	++	-	-
Cognitive impairments	++	-	?
Emotional dysregulation	++	-	-
Sleep disorder	++	++	?

++ = marked/severe
+ = mild or episodic
- = absent
? = unknown or unavailable
